# The First Cases of Imported Leprosy in Romania After More than Four Decades of Absence of New Cases

**DOI:** 10.3390/tropicalmed11080217

**Published:** 2026-08-03

**Authors:** Violeta Briciu, Mihaela Lupşe, Adrian Baican, Bogdan Fetica, Angela Monica Ionică, Georgiana Coroiu, Mirela Flonta, Andreea Jodal, Vladimir Filip, Oana Stan, Dana Marcu, Andrada-Luciana Lazar, Carmen Varodi, Corina Baican

**Affiliations:** 1 Department of Infectious Diseases and Epidemiology, “Iuliu Hațieganu” University of Medicine and Pharmacy, 400348 Cluj-Napoca, Romania; 2 Clinical Hospital of Infectious Diseases, 400348 Cluj-Napoca, Romania; 3 Department of Dermatology, “Iuliu Hațieganu” University of Medicine and Pharmacy, 400012 Cluj-Napoca, Romania; 4 Pathology Department, “Iuliu Hațieganu” University of Medicine and Pharmacy, 400337 Cluj-Napoca, Romania; 5 “Leon Daniello” Clinical Hospital of Pneumophtysiology, 400371 Cluj-Napoca, Romania; 6 Cluj County Public Health Department, 400158 Cluj-Napoca, Romania; 7 Department of Dermatology, County Emergency Clinical Hospital, 400012 Cluj-Napoca, Romania

**Keywords:** awareness, diagnosis, imported diseases, leprosy, *Mycobacterium leprae*

## Abstract

Although in Romania an increase in migrant population has been reported in the last years, there are no screening programs for infectious diseases implemented for migrants. We report the first two cases of leprosy (Hansen’s disease) since 1981 and the first imported ones in Romania, in two sisters originating from Indonesia, employed as massage therapists at a SPA center. Case one was diagnosed with multibacillary Hansen’s disease of mid-borderline type, and case two with paucibacillary Hansen’s disease borderline tuberculoid form based on epidemiological, clinical, histopathological and molecular diagnosis. Both patients have started multidrug therapy with dapsone, rifampicin and clofazimine provided by WHO. Both patients developed dapsone induced anemia under therapy, even though no G6PD deficiency was present, and dapsone was stopped. The clinical evolution of skin lesions was favorable under therapy, and the haemoglobin level started to increase after dapsone was stopped. This publication underlines the risk of importing rare diseases in Romania and in other European countries and the delay in diagnosis due to insufficient awareness among occupational medicine doctors. Improved strategies are needed at national level in order to increase awareness on rare imported infectious diseases and introduce screening programs in migrant population from high-risk areas.

## 1. Introduction

Leprosy (Hansen’s disease) is an infectious disease that still occurs in most countries worldwide, despite a steadily declining incidence of new cases almost everywhere. During 2024, a total of 172,717 new cases were reported globally, the number being 5.5% lower compared to 2023, but new cases were reported from all six WHO regions [1]. However, in the European region, there were 97 cases reported in 2024, with an increase of 162.2% compared to the previous year, and 20 patients were classified as grade-2 disability. Cases detected currently in Europe are either relapsed old cases or are represented by migrants coming from endemic areas [2]. Regarding European autochthonous leprosy, a literature review identified 18 reported cases since 2000, in: Spain (seven cases), Greece (six cases), Portugal (two cases), Italy (two cases), and Germany (one case) [3].

In Romania, the last case of leprosy (Hansen’s disease) was diagnosed in 1981. Currently, 20 leprosy patients with severe sequelae that require permanent medical care are hospitalized in the Tichilești Hospital (a hospital for care of leprosy patients, but no longer a leprosarium in the old sense of the term, a place of isolation). The leprosarium from Tichilești was established in south-eastern Romania at the beginning of the 20th century (1928), when there were about 200 patients; the number has diminished to 53 in 1991, with the last new admission taking place in 1986 [4]. The multidrug therapy (MDT) developed by WHO in 1982, was introduced in Romania in 1991, and represented the decisive act in the eradication of the disease in the country.

In the last years, an increase in migrant population in Romania has been reported, especially from Asia, as employees in different industry sectors [5]. South-East Asia Region accounted for 72% of world leprosy cases in 2024 (124,295), and Indonesia reported 14,698 cases [6].

Herein, we report the first two cases of leprosy (Hansen’s disease) since 1981 in Romania, in two sisters originating from Indonesia (Bali), employed as massage therapists at a SPA center in Cluj-Napoca, a city in North-Western Romania.

## 2. Case Description

### 2.1. Case 1

A 21-year-old patient, originating from Indonesia (Bali) and working as a massage therapist in a SPA center in Cluj-Napoca, Romania, since February 2025, with no known pathological history, presented an insidious onset of symptoms in March 2022 with paresthesia, itching and pain in the lower limbs. In 2023, she noticed the appearance of a skin lesion on her left knee, a round lesion with erythematous margins and a pale, itchy center. In evolution, paresthesia and the algic syndrome in the lower limbs have disappeared, but the skin lesion on the left knee has increased in size. Since May 2025, the patient has developed new cutaneous lesions with a similar appearance and associated pruritus on the right upper limb, the right thigh, and the posterior thorax. The patient’s mother was diagnosed with *Mycobacterium leprae* infection in Bali on 24 October 2025. Following her mother’s diagnosis, the patient self-addressed on 27 November 2025 in the outpatient clinic of the Clinical Hospital of Infectious Diseases Cluj-Napoca, from where she was addressed in the Dermatology Department of Cluj County Emergency Hospital, with the clinical and epidemiological suspicion of Hansen’s disease.

**Figure 1:**
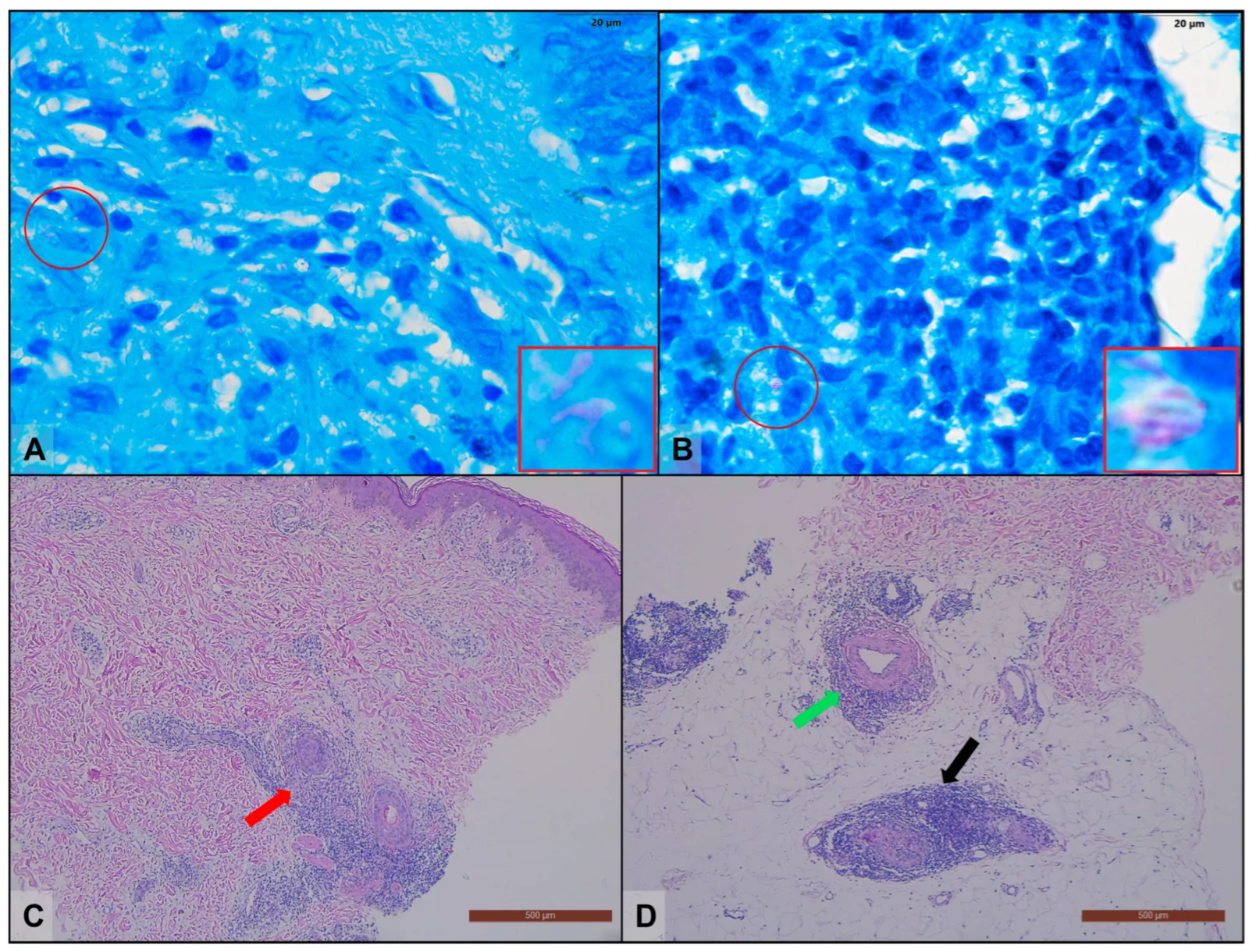
(**A**,**B**) Acid-fast bacilli present in the skin biopsy, Case1, revealed by modified Ziehl-Neelsen (Fite-Faraco) staining (circled in red and with magnification in the red square). (**C**,**D**) Histological sections of the skin biopsy performed in Case 2, revealing peri-adnexal (red arrow), peri-neural (black arrow), and peri-arteriolar (green arrow) inflammatory infiltrate.

A skin biopsy was performed on the left knee on 27 November 2025, revealing: abundant granulomatous inflammatory infiltrate arranged peri-adnexal, peri-vascular and peri-neural throughout the thickness of the dermis and acid-fast bacilli (AFB) with morphology compatible with *Mycobacterium leprae* in the modified Ziehl-Neelsen (Fite-Faraco) stain (Figure 1A,B). Fluorescence microscopy with Auramine-Rhodamine staining was also performed, that highlights AFB 3/100 field, while the PCR test for *M. tuberculosis* (Xpert^®^ MTB/RIF Ultra, Cepheid, Sunnyvale, CA, USA) was negative.

Considering the clinical, biological and epidemiological data highly suggestive for *M. leprae* infection, she was admitted on 12 December 2025 to the Clinical Hospital of Infectious Diseases Cluj-Napoca for further investigations and treatment.

Epidemiological investigation: between December 2022 and October 2024 she has worked in a hotel in Turkey, she has returned to Bali for three months, and from February 2025 she has been working in Romania. She has visited her family in Bali between September–October 2025 and in Cluj-Napoca she lives with her sister (Case 2) in the same room, in an apartment with six other persons.

**Figure 2:**
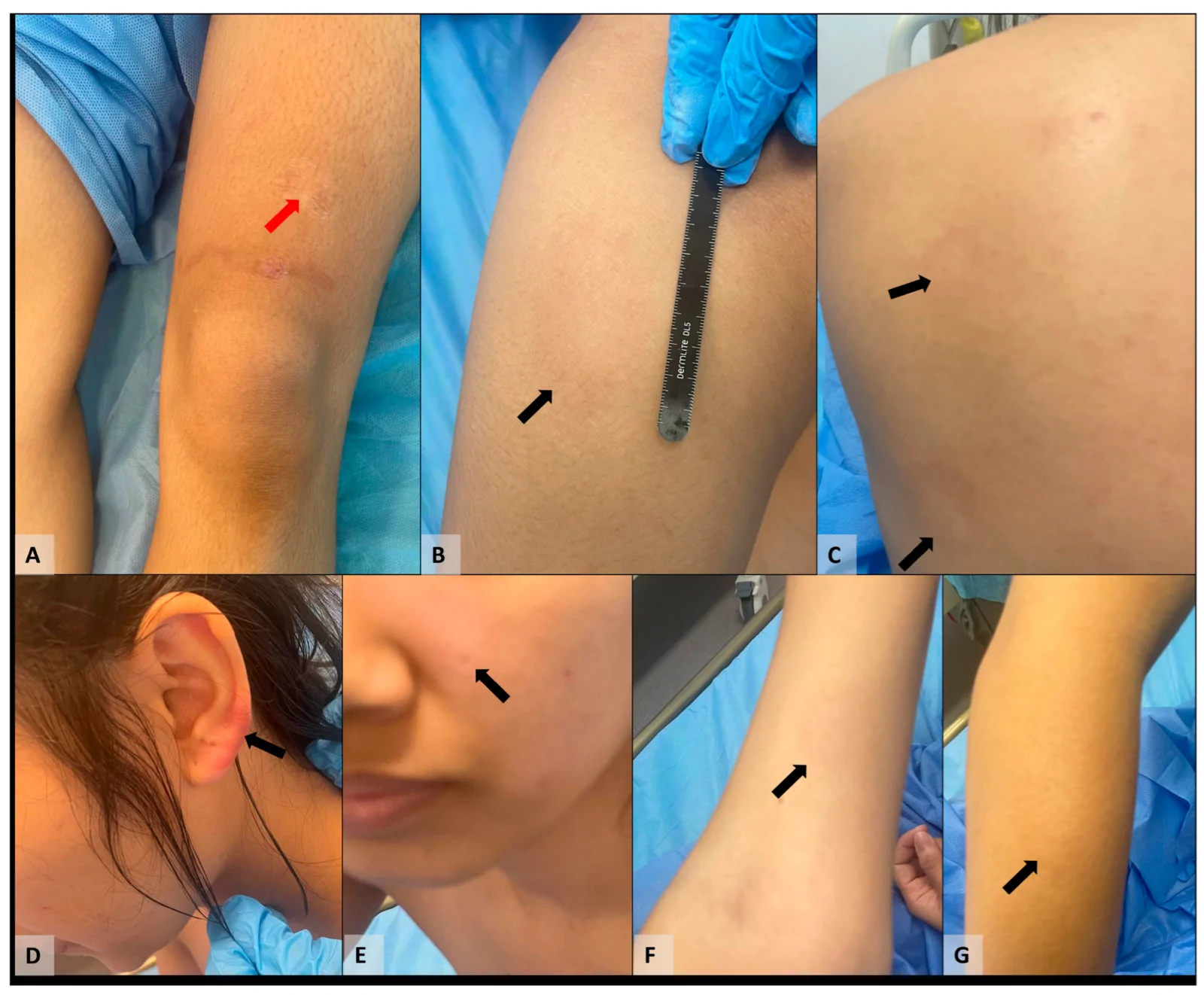
Macroscopic lesions present in Case 1: (**A**) a centrally located scar area on the anterior face of the lower left thigh which, is the place of the onset of the lesion, red arrow; erythematous lesions with annular appearance, erythematous peripheral ring and central resolution (black arrows), located on lower limbs (**B**), thorax (**C**), left ear lobe (**D**), left cheek (**E**), and upper limbs (**F**,**G**).

Clinically, the patient presented with good general condition, afebrile, while at the cutaneous-mucosal examination presented: on the upper limbs five erythematous lesions with annular appearance, erythematous peripheral ring and central resolution, no scales; on the posterior thorax, four lesions with the same characteristics, but smaller in size; on the lower limbs, nine lesions similar to those described above. The lesion located on the anterior face of the lower left thigh presented a centrally located scar area which, affirmatively, is the place of the onset of the lesion. At the level of the left earlobe a nodular erythematous lesion was noted, and on the left cheek, another erythematous lesion with slightly infiltrated periphery. Totally, 20 lesions were identified, relatively symmetrical in distribution (Figure 2).

The neurological examination (including sensory examination) revealed a global decrease in sensitivity (mainly thermal and epicritic) at the level of the lesions located on the lower and upper limbs and back; with no induration/sensitivity to palpation of peripheral nerves (ulnar, median, musculocutaneous, peroneal and tibial).

Biologically, complete blood count (CBC) and coagulation tests were within normal limits, no hepatocytolysis or cholestasis syndrome, slightly increased Indirect Bilirubin (0.3 mg/dL) (Table 1), no renal dysfunction, and no inflammatory syndrome. Copro-parasitological examination and *Giardia lamblia* antigen in stool were negative, Hepatitis B surface antigen, anti-Hepatitis C antibodies, HIV antigen/antibodies-negative. Screening for G6PD (glucose-6-phosphate dehydrogenase) deficiency was performed prior to initiation of treatment, with value within normal limits (Table 1).

There were no modifications on the EKG or pulmonary X-ray.

On 15 December 2025, the result of liquid medium culture was received which detected the growth of *Mycobacterium paragordonae*, interpreted as contamination. Solid medium culture was negative (result obtained on 26 January 2026).

**Figure 3:**
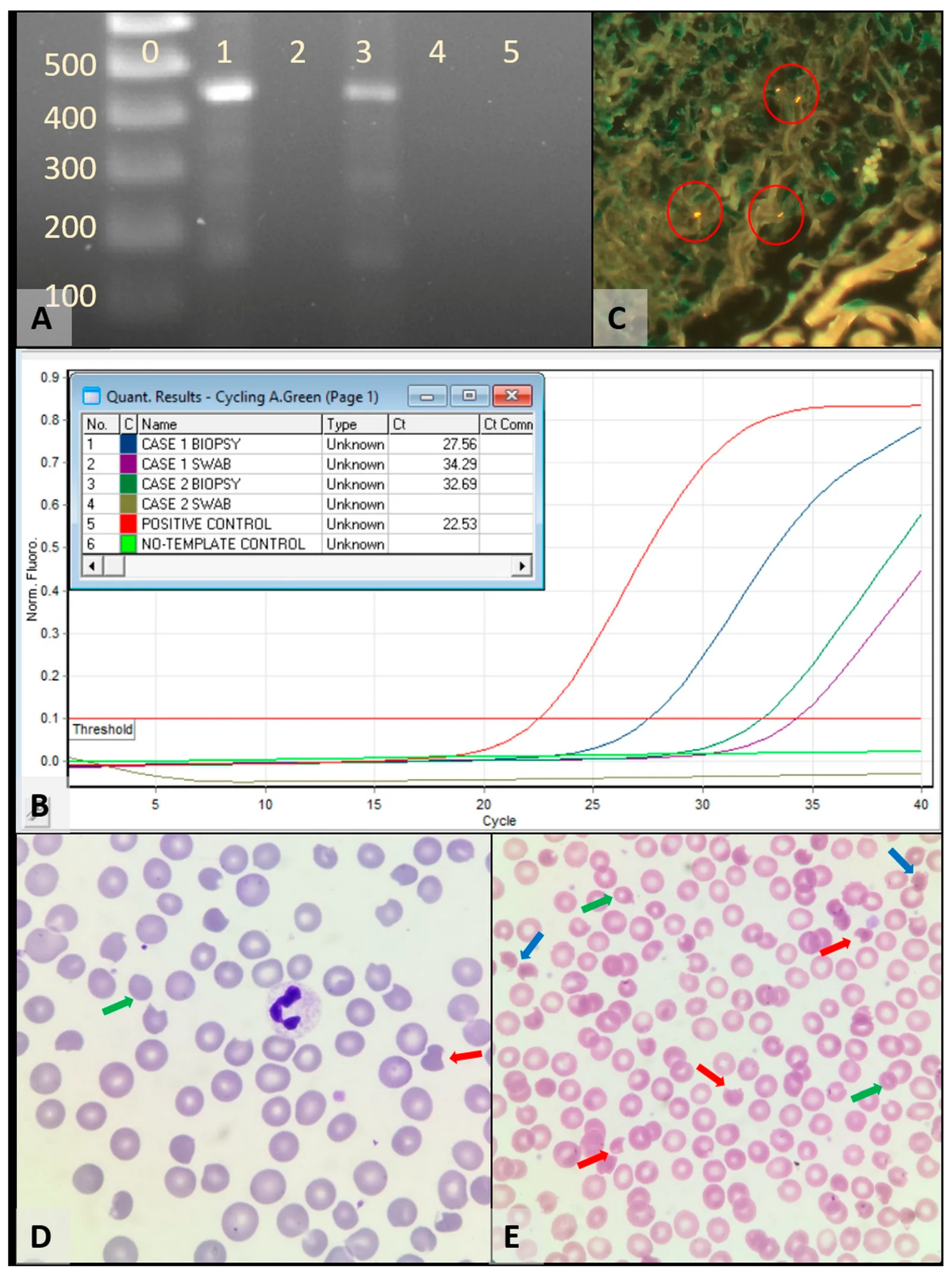
Laboratory findings in the two cases. (**A**) Electrophoretic gel, lane 0—molecular weight marker, lane 1—Case 1 biopsy, lane 2—Case 1 slit-skin swab, lane 3—Case 2 biopsy, lane 4—Case 2 slit-skin swab, lane 5—no-template control. (**B**) Real-Time PCR amplification curves. (**C**) *M. leprae* bacilli visible in Auramine-Rhodamine staining performed on histological sections (circled in red) positive 1–2 BAAR/100 fields. (**D**,**E**) Blood smears depicting schistocytes (e.g., blue arrows), microcytes (e.g., green arrows), and “bite cells” (e.g., red arrows).

On the same date, it was decided to perform a new skin biopsy and a slit-skin swab for molecular diagnosis of *M. leprae* in our service, using conventional PCR amplification of a ~450 bp fragment of the *M. leprae* specific repetitive element (*RLEP*) as described in literature [7], and a Real-Time PCR assay, using commercially available primers, probes, and a synthetic positive control (Mix 225, Omnigen, Romania). While by means of conventional PCR, only the biopsy sample was positive (Figure 3A), the Real-Time PCR revealed positivity in both samples (Figure 3B).

**Figure 4:**
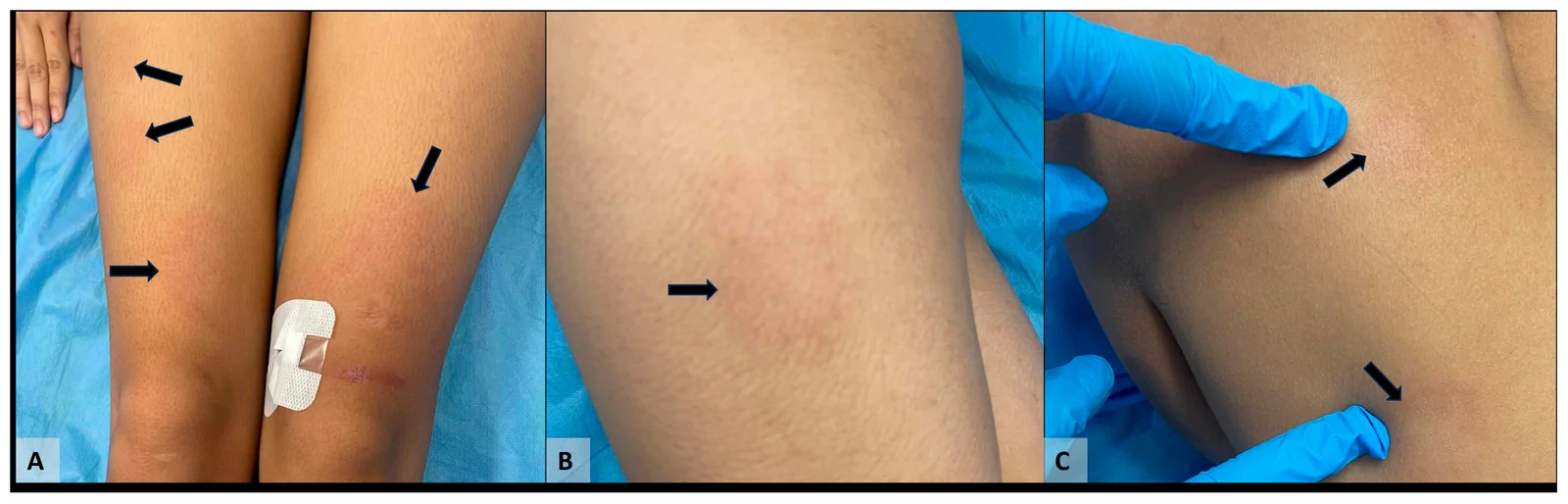
An intensification of the erythema (black arrows) in Case 1 after the first day of treatment: lower limbs (**A**,**B**), and thorax (**C**).

According to the Ridley-Jopling classification [8], the diagnosis was multibacillary Hansen’s disease of mid-borderline type (BB). According to the WHO treatment guideline [9], on the 16th of December the patient started MDT with Rifampicin 600 mg single dose (monthly) + Clofazimine 300 mg single dose (monthly), then Clofazimine 50 mg/day + Dapsone 100 mg/day, therapy kindly supported by WHO, as it was not available in Romania. In the first days of treatment the patient presented the lesions with a more erythematous aspect (Figure 4) and experienced the appearance of two new lesions (one at the level of the anterior face of the left calf and one at the level of the right breast), subsequently with favorable clinical evolution.

On 29 December the patient presented no inflammatory syndrome or hepatocytolysis, but a decrease in the level of haemoglobin (Hb) by 1.8 g/dL, and an increase in the level of Indirect Bilirubin by 0.5 mg/dL as compared to the values at hospitalization, LDH within normal limits, low haptoglobin, the blood smear with schizocytes, increased reticulocytes (Table 1). These results were interpreted as haemolysis under treatment with Dapsone (known adverse event to therapy).

The patient was discharged on the 29 December 2025 (the 14th day of MDT treatment) and returned for follow-up on the 8 January 2026. She appreciated the decrease of paresthesia in the central area of the lesions on the lower limbs, thoracic and upper limbs, accused dyspnea on moderate effort, denied dizziness, nausea or lack of appetite. The skin lesions had an improved appearance: more depigmented but without reduction in size or number, no marginal erythema, central paresthesia improved, heart rate = 110/min, EKG with sinus rhythm. Paraclinical: moderate regenerative haemolytic anemia with haemoglobin = 9.7 g/dL (decrease with 4.6 g/dL compared to the examination on 16 December, first day of MDT), increased reticulocytes, low haptoglobin level, increased LDH, mild hepatocytolysis, mild biliary retention based on indirect bilirubin (Table 1). Blood smear with anisopoikilocytosis with normocytes, schizocytes, microcytes and “bite cells” (Figure 3D). It was interpreted as post-Dapsone haemolytic anemia, therefore, it was decided to discontinue the administration of Dapsone on the 24th day of treatment and continue the MDT medication with Rifampicin and Clofazimine.

A re-evaluation was performed on the 12th of January (the last day from the first month of MDT). She denied dizziness, dyspnea, nausea, or lack of appetite. The skin lesions were much more pale, hardly perceptible in the posterior thorax, right thigh, left calf, and upper limbs, with no marginal erythema, persistence on the left knee and left ear, and improved central hypoesthesia. Paraclinical: moderate regenerative haemolytic anemia, improved compared to previous check-up, increased reticulocytes, low haptoglobin (<8 mg/dL), LDH slightly decreased compared to previous evaluation, normal transaminase level, Indirect Bilirubin with the same value as on 8th of January (Table 1), absence of bilirubin in the urine. Blood smear indicated erythrocyte anisopoikilocytosis with normocytes, schizocytes, microcytes and “bite cells”.

### 2.2. Case 2

A 25-year-old female, sibling of Case 1, originating from Indonesia (Bali) and working as a massage therapist in a SPA center in Cluj-Napoca, Romania, since February 2025, with no known pathological history, presented an insidious onset of symptoms in July 2023 with paresthesia, itching and stinging pain in the upper limbs and in the right lower limb. In 2024 she noticed the appearance of an itching erythematous skin rash on the right sole. In evolution, paresthesia and algic syndrome have diminished, but the plantar lesion has extended to the lateral face of the right leg and has been associated since 2025, with a new lesion with a similar appearance and pruritic character, on the left elbow. Following her mother’s *M. leprae* infection diagnosis, the patient and her sister (Case 1) self-addressed on 27 November 2025 for consultation.

A skin biopsy was performed from the right sole, revealing a moderate granulomatous inflammatory infiltrate arranged peri-adnexal, peri-vascular and peri-neural is detected throughout the thickness of the dermis, with no AFB in modified Ziehl-Neelsen (Fite-Faraco) stain. In the epidemiological, clinical and morpho-pathological context the suspicion of infection with *M. leprae* was raised and she was admitted on the 12th of December in the Clinical Hospital of Infectious Diseases Cluj-Napoca, together with Case 1.

Epidemiological investigation: Between August 2022 and June 2024 she has worked in a SPA in Russia, she has returned to Bali until October 2024, and starting from October 2024 she has been working in Romania. She has visited her family in Bali between September–October 2025 and in Cluj-Napoca she lives with her sister (Case 1) in the same room, in an apartment with six other persons.

**Figure 5:**
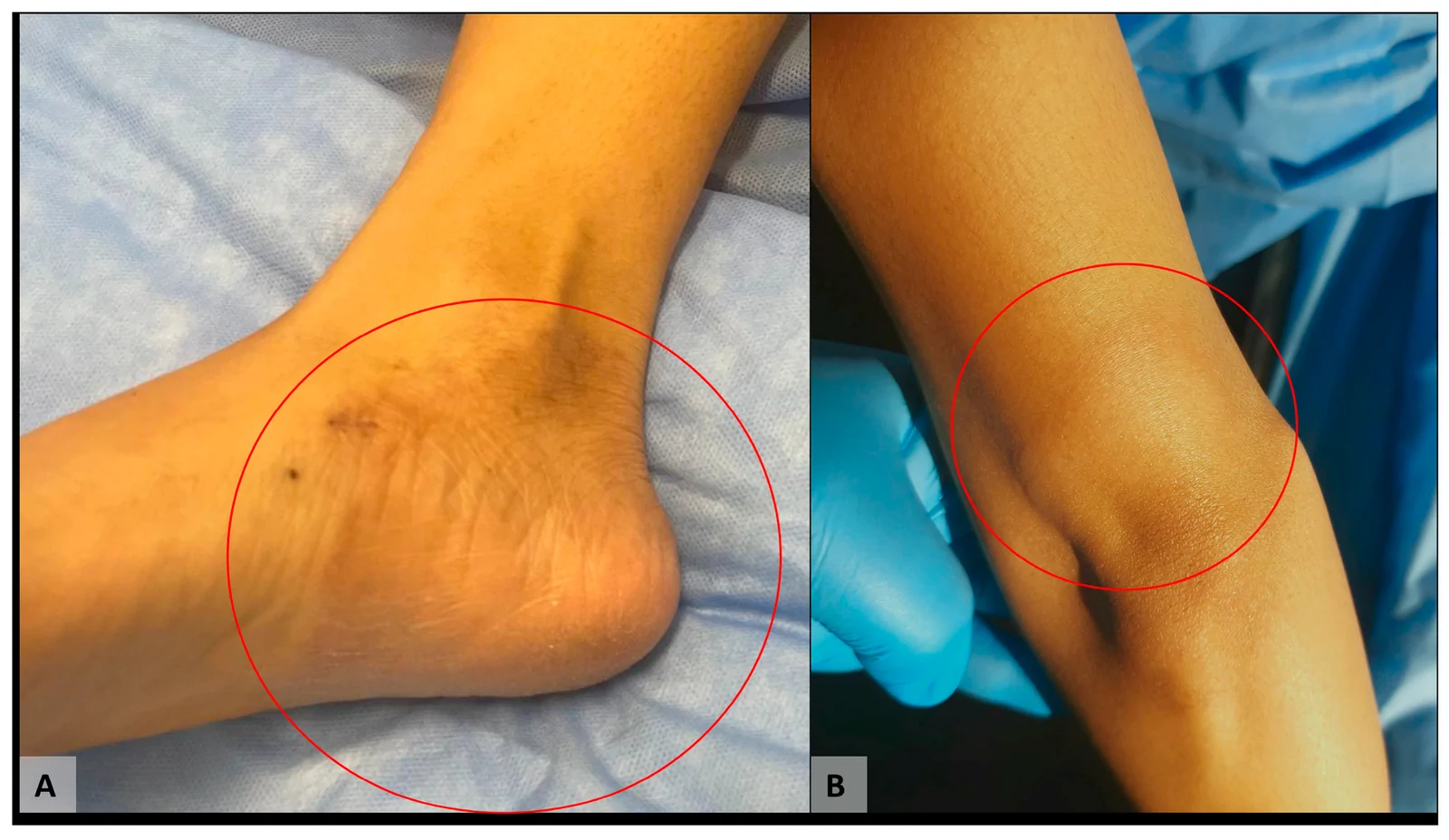
Macroscopic lesions present in Case 2 (circled in red): (**A**) circumferential lesion, with slightly elevated erythematous edges, hypopigmented in the center, ~6 cm in diameter on the right lower limb; (**B**) round macular lesion, with a diameter of 3–4 cm, a hypopigmented center and erythematous edges on the left upper limb.

At admission the patient has presented with good general condition, afebrile, while the physical exam revealed: on the right lower limb (plantar and lateral face of the foot), one circumferential lesion, with slightly elevated erythematous edges, hypopigmented in the center, ~6 cm in diameter, with central hypoesthesia; on the left upper limb, a macular lesion, round with a diameter of 3–4 cm, a hypopigmented center and erythematous edges, without central hypoesthesia (Figure 5).

Paraclinical: complete blood count (CBC) and coagulation tests in normal range, no hepatocytolysis or cholestasis syndrome, minimally increased Indirect Bilirubin (Table 2), no renal dysfunction, no inflammatory syndrome. Copro-parasitological examination and *Giardia lamblia* antigen in stool were negative, HBV antigen, anti-HCV antibodies, and HIV antigen/antibodies were negative. Screening for G6PD deficiency was performed prior to initiation of treatment, with values within normal limits (Table 2). No modification on EKG or pulmonary X-ray. Neurological examination: symmetrical osteotendinous reflexes, no muscle strength deficit, hypoesthesia at the level of the lesion on the foot (thermal, algic hypoesthesia, fine/epicritic sensibility), proprioception preserved at the level of the four limbs. No sensitivity to palpation of peripheral nerves (ulnar, median, musculocutaneous, peroneal and bilateral tibial).

On 15 December 2025, the result of the culture on liquid media (from 27 November 2025) detected the growth of *M. paragordonae*, interpreted as contamination. Solid medium culture was negative (result obtained on 26 January 2026).

A slit-skin at the level of the earlobe was performed on the 15 December, with direct microscopy in Ziehl-Neelsen stain and fluorescence in Auramine-Rhodamine staining negative, and a skin biopsy from the second lesion (left upper limb) was repeated for histopathological examination showing a fragment of orthokeratotic skin, presenting at the dermis level nodular foci poorly delimited by granulomatous inflammation with epithelioid cells and lymphocytes, without necrosis. They were predominantly arranged in the deep dermis and in the hypoderm, peri-adnexal, peri-neural and peri-arteriolar (Figure 1C,D). A few small granulomas were also identified at the level of the superficial, perivascular dermis. The modified Ziehl- Neelsen (Fite-Faraco) stain for mycobacteria was negative. Auramine-Rhodamine staining performed on histological sections was positive 1–2 BAAR/100 fields (Figure 3C). Conclusion: the microscopic appearance corresponds to cutaneous granulomatosis; in the clinico-epidemiological context, the histological appearance may correspond to a skin lesion of leprosy, the histological tuberculoid form.

The molecular diagnosis presented a positive result for *Mycobacterium leprae* from the skin biopsy and a negative result from the slit-skin sample (Figure 3A,B) using the same PCR assays as for Case 1.

The second case was interpreted as paucibacillary Hansen’s Disease Borderline Tuberculoid form according to the Ridley-Jopling classification [8]. According to the WHO guideline [9], the patient started MDT on the 16th of December, with Rifampicin 600 mg single dose (monthly) + Clofazimine 300 mg single dose (monthly), then Clofazimine 50 mg/day + Dapsone 100 mg/day, therapy provided by WHO.

Under the treatment, the patient presented a favorable clinical evolution, without the appearance of new skin lesions, but on the 14th day of treatment presented a slight decrease in the level of haemoglobin, by 1.3 g/dL and an increase in the level of Indirect Bilirubin by 0.5 g/dL, compared to the values at hospitalization, slightly increased reticulocytes and LDH within normal limits, haptoglobin with low value (Table 2), the blood smear with rare schizocytes, interpreted in the context of haemolysis under treatment with Dapsone. The patient was discharged on the 29 December 2025 (the 14th day of treatment) and returned for follow-up on the 8 January 2026. She described the amelioration of paresthesia in the central area of the lesion on the lower right limb and denied dizziness, dyspnea, nausea, or lack of appetite.

On clinical examination, the patient was afebrile, with a heart rate of 115/min and sinus rhythm on EKG. The skin lesions had an improved appearance, more depigmented, without marginal erythema, the lesion on the left elbow was hardly visible, central paresthesia affirmatively relieved. Paraclinical: moderate regenerative haemolytic anemia syndrome with haemoglobin = 11.2 g/dL (decrease of 4.1 g/dL in dynamics compared to the examination on December 16th, the first day of MDT) (Table 2). Blood smear indicated erythrocyte anisopoikilocytosis with normocytes, microcytes and “bite cells”, schizocytes (Figure 3E).

She had increased reticulocytes, mild biliary retention based on the indirect component (Table 2). It was interpreted as post-drug haemolytic anemia in the context of Dapsone administration, therefore it was decided to discontinue the administration of Dapsone on the 24th day of treatment and to continue the MDT medication with Rifampicin and Clofazimine.

She returned for follow-up on the 12th of January (the last day from the 1st month of MDT). She denied dizziness, dyspnea, nausea or lack of appetite. The skin lesions had an improved appearance: they were more pale, central hypoesthesia affirmatively improved, the lesion on the left elbow is hardly visible Paraclinical: moderate regenerative haemolytic anemia with haemoglobin = 11.2 g/dL (stationary compared to 8 January), increased reticulocytes, low haptoglobin, increased LDH (239 U/L), slight biliary retention based on the indirect component, slightly increasing compared to the previous examination (Table 2), the urine test without bilirubinuria. Blood smear indicated erythrocyte anisocytosis with normocytes, microcytes, “bite cells” and schizocytes.

Both patients left Romania on the 14 January 2026, to fly back to Bali intending to follow-up the treatment and be monitored by the physician in charge for their mother. They completed a declaration that they will self-address for monitoring in Bali and they received the medication provided by WHO for the entire treatment, excluding Dapsone, due to haemolytic anemia.

An epidemiological investigation for contact tracing was made by the local public health department. Seven contacts were identified based on the WHO definition (any person who had been in contact with the untreated index case for at least 20 h per week, for at least 3 months in the preceding year) [9], respectively six colleagues from work who lived in the same apartment and the manager of the SPA. All were evaluated clinically in our department, none presented any skin lesion or modification on neurological examination excluding leprosy and none presented manifestations of tuberculosis; they all have received chemoprophylaxis with Rifampicin 600 mg, according to WHO [10].

## 3. Discussion

We presented the first two imported cases of leprosy and the first cases diagnosed with this condition in Romania in 44 years. An increase in the number of imported cases of rare infectious diseases is to be expected due to a high immigration rate from high-risk regions. In Romania there are no screening programs implemented for migrants. ECDC has provided in 2018 Public health guidance on screening and vaccination for infectious diseases in newly arrived migrants within the EU/EEA [11], including recommendations for active and latent tuberculosis, HIV, hepatitis B and C, schistosomiasis and strongyloidiasis, and vaccine preventable diseases. For leprosy, a complete clinical examination with focus on cutaneous lesions, neurological symptoms and any epidemiological link are important elements to be performed by a clinician, in order to have early diagnosis for treatment or prophylaxis in contacts. There is no test recommended to diagnose leprosy infection (latent leprosy) among asymptomatic contacts [10]. In Romania, a medical examination is performed by an occupational medicine doctor at the time of employment, but it is important to have increased knowledge for suspicion on rare imported infectious diseases. Our patients were evaluated at employment, and although the skin lesions were present by that time, no clinical suspicion was raised. It should be noted also that the patients have been working in other countries (Turkey, Russia) since the debut of symptomatology and cutaneous lesions, and no clinical suspicion was raised by any occupational medicine doctor. The patients self-addressed for an infectious diseases consultation, due to their mother’s confirmation with leprosy. In most cases reported in Europe, there is a diagnostic delay due to low physician awareness of the disease, the epidemiological link being very important [3].

Despite significant advances in the treatment of leprosy, gaps in knowledge regarding disease transmission, clinical picture, management, side effects of medication and antibiotic resistance represent continuing challenges [12]. The 2018 WHO guidelines recommend treatment with rifampicin, dapsone, and clofazimine for paucibacillary and multibacillary leprosy for 6 and 12 months, respectively [8]. Laboratory monitoring includes baseline laboratory values in addition to trending blood counts and liver function tests to evaluate for side-effects of medications. Side-effects attributed to MDT are more frequent than previously described, resulting in interruption of treatment in many patients, with dapsone being most frequently involved [13,14,15].

Dapsone (4,4-diamino-diphenyl sulfone) inhibits bacterial synthesis of dihydrofolic acid and major adverse effects are represented by methaemoglobin formation, haemolysis and agranulocytosis. Dapsone-induced haemolysis is common, and individuals with a deficiency of erythrocytic glucose-6-phosphate dehydrogenase (G-6-PD) enzyme are more susceptible [16]. However, acute haemolysis can also occur in persons with normal G-6-PD activity, as in our patients. A 5-year retrospective study on 171 patients that received treatment for leprosy in India identified dapsone-induced haemolysis in 12 patients (7%) and dapsone was substituted with clofazimine in paucibacillary disease and ofloxacin in multibacillary disease [17]. Alternate regimens suggested by the National Hansen’s Disease Program may include minocycline (100 mg/day) as an alternative for dapsone [18].

As both patients left the country and returned to Bali for treatment continuation on the 30th day of treatment, no substitution of dapsone was decided in our center, leaving the decision to physicians in Bali, based on drugs availability (ofloxacin or minocycline as possible options).

## 4. Conclusions

This publication underlines the risk of importing rare diseases in Romania and in other European countries where autochthonous disease is rare or absent and the delay in diagnosis due to insufficient awareness among occupational medicine doctors. Improved strategies are needed at national level in order to increase awareness on rare imported infectious diseases and introduce screening programs in migrant population from high-risk areas.

## Figures and Tables

**Table 1:** Paraclinical examination results in Case 1.

Analyte	Normal Range	12 December 2025	16 December 2025	21 December 2025	29 December 2025	8 January 2026	12 January 2026
G6PD	7.9–16.3 U/gHb	10.2					
Hb	11.5–15.4 g/dL	13.5	14.3	12.8	11.7	9.7	10.3
haptoglobin	35–250 mg/dL				<8	<8	<8
BD	0–0.5 mg/dL	0.2	0.2	0.4	0.5	0.6	0.6
BI	0–0.09 mg/dL	0.3	0.3	0.7	0.8	1.1	1.1
TGP	0–34 U/L	17	13	9	12	36	23
LDH	125–220 U/L				213	329	317
reticulocytes	up to 0.075 × 10^3^/μL				0.1338 × 10^3^	0.2215 × 10^3^	0.2967 × 10^3^

G6PD—glucose-6-phosphate dehydrogenase; Hb-haemoglobin, BD—Direct Bilirubin, BI—Indirect Bilirubin, TGP—Alanine aminotransferase, LDH—Lactate Dehydrogenase.

**Table 2:** Paraclinical examination results in Case 2.

Analyte	Normal Range	12 December 2025	16 December 2025	21 December 2025	29 December 2025	8 January 2026	12 January 2026
G6PD	7.9–16.3 U/gHb	10.4					
Hb	11.5–15.4 g/dL	14.7	15.3	15.2	13.4	11.2	11.2
haptoglobin	35–250 mg/dL				<8		<8
BD	0–0.5 mg/dL	0.2	0.2	0.3	0.4	1.4	0.6
BI	0–0.09 mg/dL	0.4	0.3	0.6	0.9	0.8	1.1
TGP	0–34 U/L	29	17	10	10	22	15
LDH	125–220 U/L				196		239
reticulocytes	up to 0.075 × 10^3^/μL				0.1984 × 10^3^	0.3151 × 10^3^	0.4084 × 10^3^

G6PD—glucose-6-phosphate dehydrogenase; Hb-haemoglobin, BD—Direct Bilirubin, BI—Indirect Bilirubin, TGP—Alanine aminotransferase, LDH—Lactate Dehydrogenase.

## Data Availability

All relevant data and materials used during this study are enclosed within the article.

## Abbreviations

The following abbreviations are used in this manuscript:

AFB	Acid-Fast Bacili
BD	Direct Bilirubin
BI	Indirect Bilirubin
CBC	Complete Blood Count
G6PD	Glucose-6-Phosphate Dehydrogenase
Hb	Haemoglobin
LDH	Lactate Dehydrogenase
TGP	Alanine aminotransferase
PCR	Polymerase Chain Reaction
